# Antibody Targeting Soluble NKG2D Ligand MIC Enhances Adoptive T-Cell Therapy in an NK Cell-Dependent Mechanism

**DOI:** 10.3390/biomedicines14102390

**Published:** 2026-10-19

**Authors:** Fahmin Basher, Tyler A. Smith, Mark P. Rubinstein, Jennifer D. Wu

**Affiliations:** 1 Division of Hematologic Malignancies and Cellular Therapy, Department of Medicine, Duke University School of Medicine, Durham, NC 27710, USA; fahmin.basher@duke.edu; 2 Department of Microbiology and Immunology, Medical University of South Carolina, Charleston, SC 29425, USA; mark.rubinstein@osumc.edu; 3 Department of Urology, Feinberg School of Medicine, Northwestern University, Chicago, IL 60611, USA; tyler.smith128@northwestern.edu; 4 Division of Medical Oncology, Department of Internal Medicine, The Ohio State University, Columbus, OH 43210, USA; 5 Pelotonia Institute for Immuno-Oncology, The Ohio State University, Columbus, OH 43210, USA

**Keywords:** antibody, NKG2D, soluble MIC (sMIC), adoptive T cell therapy, NK cells

## Abstract

**Background/Objectives:** Antigen-specific adoptive CD8^+^ T-cell therapy has demonstrated promising clinical activity in selected cancers; however, its broader efficacy against solid tumors remains limited by the immunosuppressive tumor microenvironment (TME), which compromises the persistence and function of transferred T-cells. A clinically relevant component of the human solid-tumor TME is soluble MHC class I chain-related molecules A and B (sMICA and sMICB, collectively termed sMIC), which are tumor-derived soluble NKG2D ligands that are not naturally expressed in mice. sMIC promotes tumor immune evasion through multiple mechanisms, but whether therapeutic clearance of sMIC can enhance the efficacy of adoptive T-cell therapy remains unclear. **Methods:** B16F10 melanoma cells engineered to express soluble MICB (B16F10-sMICB) were implanted subcutaneously into syngeneic B6/MICB mice. Once tumors became palpable, mice were randomized to four treatment groups: (1) no treatment; (2) adoptive transfer of gp100-specific Pmel CD8^+^T cells; (3) the sMIC-targeting monoclonal antibody B10G5; or (4) B10G5 combined with Pmel adoptive cell transfer. Pmel CD8^+^T cells were administered once intravenously, and B10G5 was administered intraperitoneally twice weekly at approximately 3 mg/kg. Tumor growth and body weight were monitored twice weekly. To determine the contribution of natural killer (NK) cells to the therapeutic effect of the combination treatment, NK cells were depleted using the anti-NK1.1 antibody PK136. The impact of NK cells on CD8^+^T cell function during B10G5/sMIC stimulation was further evaluated ex vivo. **Results:** Therapeutic targeting of sMIC with B10G5 significantly enhanced the antitumor efficacy of adoptively transferred Pmel CD8^+^T cells and was associated with increased donor T-cell persistence, tumor infiltration, and antigen-specific effector function. NK cell depletion markedly diminished the antitumor efficacy of the B10G5/Pmel combination and reduced the abundance, activation, and effector function of donor CD8^+^ T-cells. Consistent with these in vivo findings, ex vivo depletion of NK cells impaired IFN-γ production by NKG2D^+^CD8^+^ T-cells during B10G5/sMIC stimulation, supporting a functional role for NK cells in sustaining CD8^+^T cell responses. **Conclusions:** These findings provide evidence that therapeutic targeting of tumor-derived sMIC can enhance the efficacy of antigen-specific adoptive CD8^+^T cell therapy by promoting the persistence and function of transferred T-cells. Importantly, our results identify NK cells as critical contributors to the enhanced therapeutic response, revealing functional cooperation between NK cells and adoptively transferred CD8^+^T cells during sMIC-targeted therapy. Together, these findings provide preclinical proof-of-concept for combining sMIC-targeting antibodies with adoptive T-cell therapy to improve antitumor immunity against sMIC^+^ solid tumors.

## 1. Introduction

Despite recent advances in adoptive T-cell therapy (ACT) strategies, with recent FDA approvals of tumor-infiltrating lymphocytes (TILs) in metastatic melanoma and T-Cell Receptor (TCR) engineered T-cells in metastatic synovial sarcoma [1,2], ACT strategies still result in suboptimal clinical outcomes despite remarkable preclinical efficacy [3,4]. A major challenge in solid tumors is the immunosuppressive tumor microenvironment (TME) that hinders ACT persistence and function [3,4]. Factors contributing to the hostile TME include tumor-derived soluble factors that directly suppress CD8 T-cell persistence and function or indirectly suppress ACT efficacy through modulating the TME [4,5].

Current strategies to reduce immunosuppression in the tumor microenvironment include the use of checkpoint inhibitor combinations [6,7,8], TGF-β inhibitors [9,10], targeting adenosine pathways (CD39/CD73/A2A) [11], targeting CSF1R to inhibit the activity of immune suppressive macrophages [12,13], CD47-SIRPa blockade [14], CXCR2/IL-8 inhibition to reduce neutrophil/MDSC recruitment [15], IDO/TDO inhibitors [16], anti-VEGF therapy [17], and stromal remodeling [18]. Due to the immune regulatory role of cytokines, chemokines, and associated immune receptors in normal tissue homeostasis, systemic targeting of these immunosuppressive factors to enhance ACT may elicit unwanted immune toxicity or inadvertently stimulate tumor growth through compensatory pathways, potentially undermining therapeutic outcome [19,20,21,22,23,24,25]. This is demonstrated by many efforts to target TGF-β [26]. Thus, a more tumor-specific strategy to enhance ACT for solid tumors for both safety and efficacy is needed.

The C-lectin type activating immune receptor NKG2D is constitutively expressed by all human NK cells and CD8 T-cells and serves as an activating and co-stimulatory receptor, respectively [27]. The ligands of NKG2D are not expressed by most healthy tissues but are often induced in transformed cells [27,28,29,30]. A variety of NKG2D ligands have been identified, presumably due to evolutionary pressure [30,31], among which the family members of the MHC class I chain-related molecules A and B (MICA/B, collectively termed MIC) are most prevalently expressed by human solid tumors in response to genotoxic stress [30]. The MIC molecule was initially expressed as a membrane-bound cell surface molecule during early tumorigenesis to incite immune surveillance against abnormal cells [32,33]. However, in advanced malignancies, tumor cells shed soluble MIC (sMIC), which was initially thought to be a mechanism to evade immune surveillance [34,35,36,37]. sMIC can negatively impact tumor immunity in multiple ways, such as inducing dysfunction of CD8^+^T cells and NK cells in tumors and expanding the immune-suppressive myeloid population microenvironment [38,39,40]. Elevated serum sMIC is highly associated with the progression of multiple cancer types such as colon adenocarcinoma, prostate carcinoma, multiple myeloma, pancreatic carcinoma, and neuroblastoma [39,41,42,43,44]. Patients with elevated serum sMIC responded poorly to immune checkpoint blockade therapy [45,46]. Collectively, these data demonstrated the immunosuppressive impact of tumor-derived sMIC on tumor immunity. However, it is not known whether targeting sMIC can enhance T-cell-based therapy. In this current study, we investigated the impact of co-administration of an sMIC-targeting antibody on adoptive CD8 T-cell therapy using a well-established melanoma model. We further investigated whether NK cells impact the efficacy of combination therapy.

## 2. Materials and Methods

### 2.1. Mice and Cell Lines

Mice were bred and housed under specific pathogen-free conditions in the animal facilities at the Medical University of South Carolina and Northwestern University in accordance with institutional guidelines and approved Institutional Animal Care and Use Committee (IACUC) protocols. All mice were housed in specific pathogen-free (SPF) facilities. All mice used were in the B6 background. The MICB transgenic mice in C57/B6 background, defined as B6/MICB mice, have been previously described [47]. Pmel-1 transgenic mice are previously described elsewhere [48]. Briefly, human full-length MICB was overexpressed in the prostate of C57BL/6J (B6) mice under the control of the male hormone-sensitive rat probasin (rPB) promoter to generate B6/MICB mice [47]. Because the expression of MICB is driven gradually by male hormones in male mice, only male B6/MICB mice have MICB expression. Those mice have no phenotypical, functional, or immunological differences from WT mice [47]; however, they are more tolerant in host MIC-expressing syngeneic tumor cell lines, with less probability of generating anti-MIC autoantibodies [47]. The sMIC-expressing B16F10-sMICB cell line was generated by transduction of B16F10 cells (ATCC; Manassas, VA, USA) with an IRES-GFP retroviral vector containing the puromycin-resistant marker and the construct for recombinant secretable soluble MICB (sMICB), as described previously [49]. The control B16F10-GFP cell line was generated similarly with the same IRES-GFP retroviral vector containing the puromycin-resistant marker but without sMICB. B16F10 cells expressing sMICB were selected by puromycin in culture for five days, followed by flow cytometry sorting for GFP-positive cells. The GFP-positive control B16F10 (no sMICB) was similarly selected by puromycin, followed by flow cytometry sorting for GFP positivity. Expression of sMICB in GFP^+^ B16F10-sMICB cells was further confirmed by intracellular staining with the anti-sMIC/MIC monoclonal antibody B10G5 (Supplementary Figure S1). For simplicity, B16F10-sMICB-GFP and B16F10-GFP cell lines were referred to as B16F10-sMICB and B16F10, respectively.

### 2.2. B10G5 Antibody

Generation of the anti-MIC/anti-sMIC monoclonal antibody (mAb) B10G5 was previously described [47]. B10G5 is a non-blocking sMIC-neutralizing monoclonal antibody that neutralizes free sMIC but does not block the interaction of MIC with the receptor NKG2D [50]. B10G5 is mouse IgG1.

### 2.3. Tumor Inoculation and In Vivo Pmel ACT Experiment

B16F10-sMICB cells were implanted subcutaneously into cohorts of syngeneic B6/MICB male mice (4 × 10^5^ cells per mouse) at 8–10 weeks of age. When tumor volume reached approximately 50–100 mm^3^, animals were randomized into four therapy groups (n = 3–8 per group): (1) isotype control mouse IgG1 (cIgG) (3 mg/kg BW); (2) anti-MIC mAb B10G5 (mIgG1, 3 mg/kg BW); (3) Pmel ACT and cIgG; and (4) Pmel ACT and B10G5. cIgG and B10G5 were given intraperitoneally (i.p.) the day before Pmel adoptive transfer at a dose of 3 mg/kg and thereafter twice weekly. Pmel cells were given via tail vein (i.v.) injection at a dose of 5 × 10^6^ cells/ mouse and were only given once to animals for the duration of the experiment. For NK depletion experiments in the relevant group of animals, anti-NK1.1 antibody (PK136, BioXCell; Lebanon, NH, USA) was given i.p. twice weekly at a dose of 3 mg/kg until the study endpoint. Tumor volume of 800 mm^3^ was defined as the survival endpoint. After euthanization, spleens, tumor-draining lymph nodes, and tumors were harvested for analyses.

### 2.4. Preparation of Pmel Cells for ACT

Splenocytes were obtained from Pmel-1 mice B6.Cg-Thy1a/CyTg(TcraTcrb)8Rest/J (Jackson Laboratory; Bar Harbor, ME, USA), strain 005023, which carries a rearranged T-cell receptor transgene specific for the mouse homolog (pmel-17) of human melanoma antigen hgp100_25-33_ (gp100) and the congenic marker Thy1.1 for tracking adoptively transferred Pmel-1 cells. Pmel-1 splenocytes (referred to as Pmel cells thereafter) were cultured in the presence of 1 μM hgp100 (KVPRNQDWL, GenScript; Piscataway, NJ, USA) and 10 ng/ml of IL-12 in complete RPMI 1640 medium for 3 days before being used for ACT experiments as described elsewhere [51]. Pmel cells were transferred via lateral tail vein injection (i.v.).

### 2.5. Ex Vivo Cytokine Re-Stimulation and Cytotoxicity Assay

For assaying intracellular IFN-γ, single-cell suspensions of splenocytes and tumor-draining lymph nodes were stimulated at 37 °C for 6 h with 50 ng/mL phorbol myristate acetate (PMA) and 500 ng/mL ionomycin in the presence of BD GolgiPlug (BD; Franklin Lakes, NJ, USA). Cells were harvested and analyzed by flow cytometry for intracellular IFN-γ. For cytotoxicity assays, we used CD107a as a marker for cellular degranulation in response to re-stimulation. For antigen-specific T-cell function, single-cell suspensions of bulked splenocytes or tumor-draining lymph nodes were stimulated with 1 μM of melanoma antigen gp100_25−33_ overnight, followed by addition of anti-CD107a antibody and BD GolgiStop for an additional 4 h before flow cytometry analysis.

### 2.6. ELISpot Cytokine Assay

Single-cell suspensions of splenocytes from B16F10-sMICB-challenged B6/MICB mice treated with either Pmel ACT in combination with B10G5, or Pmel ACT in combination with cIgG, were seeded on 96-well pre-coated mouse IFN-γ ELISpot plates (ELISpot Plus: Mouse IFN-γ (HRP), Mabtech, Cat# 3321-4HPW-2; Cincinnati, OH, USA). Cells were stimulated with 1μM gp100_25−33_, and the plates were wrapped with foil and incubated at 37 °C overnight. Unstimulated samples were used as negative controls. Plates were then labeled with 1 μg/mL anti-mouse IFN-γ-biotin for 2 h, followed by streptavidin-HRP for 1 h, then TMB substrate for 15 min according to manufacturer guidelines. Spots were imaged using a dissection microscope and counted using ImageJ software v1.49.

### 2.7. Ex Vivo NK Cell Depletion Assay

For ex vivo NK cell depletion, single-cell suspensions of splenocytes from adult wild-type B6 mice were incubated with 0.03 μg/mL biotinylated anti-NK1.1 (clone PK136, Biolegend, Cat# 108704; San Diego, CA, USA) for 15 min on ice, then washed with MojoSort Buffer (Biolegend, Cat# 480017). Cells were then incubated with MojoSort Streptavidin nanobeads (Biolegend, Cat# 480016) for 15 min on ice, followed by washing with MojoSort Buffer. NK-depleted splenocytes were negatively sorted using a 5 mL MojoSort magnet (Biolegend, Cat# 480019). The depletion efficiency was confirmed by flow cytometry analysis. NK cell-depleted or non-depleted splenocyte cultures were seeded at 1 × 10^6^ cells per well of a 24-well plate in 1 mL RPMI 1640 supplemented with 10% FBS and 1% penicillin–streptomycin. Cells were then stimulated with 1 μg/mL plate-bound anti-mouse CD3 antibody (clone 145-2C11, BD, Cat# 553057), 1 μg/mL anti-mouse CD28 antibody (clone 37.51, BD, Cat# 553294), and 100 ng/mL of B10G5 complexed with recombinant human MICB His-tag (Sino Biological, Cat# 10759-H08H; Paoli, PA, USA) (B10G5/sMICB complex, pre-mixed for 30 min at room temperature) and cultured for 3 days before analysis via flow cytometry.

### 2.8. Flow Cytometry Analysis

Single-cell suspensions were incubated on ice for 30 min with a combination of antibodies specific to cell surface markers for identifying lymphocyte subsets. These antibodies are: anti-NK1.1 (clone PK136), anti-NKp46 antibody (clone 29A1.4), anti-CD3ε (clone 145–2C11), anti-CD8a (clone 53–6.7), anti-CD4 (clone RM4–5), anti-NKG2D (clone CX5), anti-CD44 (clone IM7), anti-CD25 (clone PC61), anti-CD62L (clone MEL-14), anti-CD90.1 (Thy1.1) (clone S20007C), anti-CD107a (clone 1D4B), and anti-IFN-γ (XMG1.2). All antibodies were from BioLegend. For intracellular staining, cells were stained with surface markers followed by fixation and permeabilization with CytoFix/CytoPerm kits (BD) and antibodies specific to intracellular molecules. Cells were analyzed using a LSR Fortessa flow cytometer (BD). Data were analyzed using FlowJo v10 software (Tree Star; Ashland, OR, USA).

### 2.9. Statistical Considerations

Tumor growth curves were analyzed via a mixed-effects model followed by Šídák’s multiple comparisons test. Donor Pmel-1 cell frequencies in the circulation tracked across multiple days were analyzed via two-way repeated-measures ANOVA, followed by Šídák’s multiple comparisons test. Simple differences between matched or unmatched means of two populations were compared by paired or unpaired two-tailed *t*-tests (Gaussian distribution), or by Mann–Whitney U test (unpaired, non-Gaussian distribution) or Wilcoxon matched-pairs signed-rank test (paired, non-Gaussian distribution). Survival was determined via Kaplan–Meier analysis, with comparison of curves using the log-rank (Mantel-Cox) test. A *p* value of less than 0.05 was considered significant. GraphPad Prism version 11 (GraphPad Software, Boston, MA, USA) was used for all analyses. * *p* < 0.05, ** *p* < 0.01, *** *p* < 0.001, **** *p* < 0.0001, ns = not significant (*p* ≥ 0.05).

## 3. Results

### 3.1. Therapeutic Targeting of sMIC with B10G5 Enhances the Antitumor Efficacy of Pmel ACT in Mice Bearing sMIC-Producing Tumors

**Figure 1:**
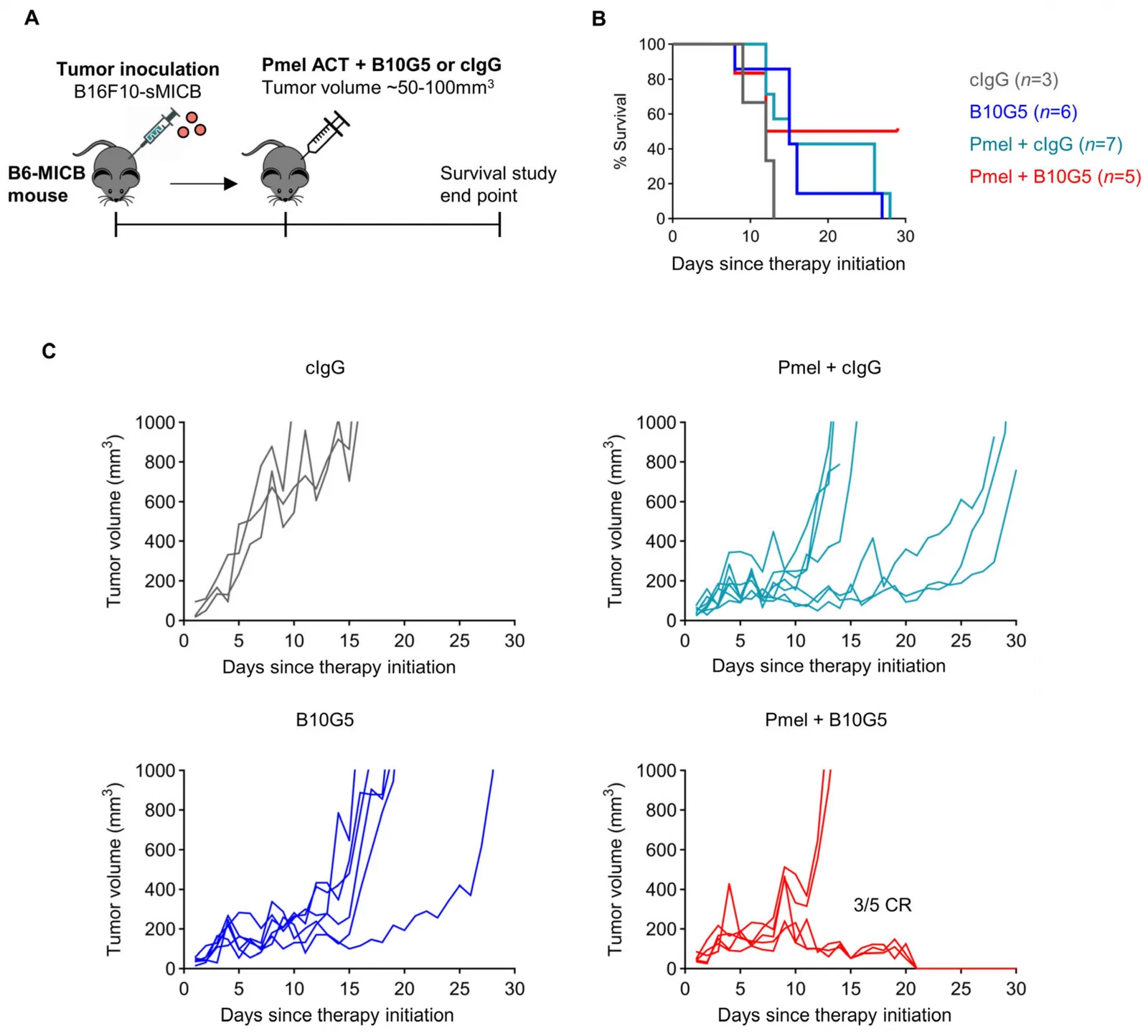
Enhanced Pmel ACT therapeutic effect with antibody B10G5 targeting sMIC. (**A**) Experimental design. B6/MICB male mice were subcutaneously inoculated with 4 × 10^5^ B16F10-sMICB cells. When tumors were established to a volume of 50–100 mm^3^, mice were randomized into 4 treatment groups as indicated. Isotype control mouse IgG1 (cIgG) and B10G5 were given i.p. one day before Pmel ACT and thereafter twice weekly at a dose of 3 mg/kg during experiments. Pmel ACT (5 × 10^6^ cells/mouse) was given i.v. tail vein injection once only during experiments. (**B**) Kaplan–Meier survival curve of B16F10-sMICB tumor-bearing mice that received 4 different therapies as indicated. (**C**) Individual tumor growth curves in each therapy group. CR, complete tumor regression.

To determine whether therapeutic targeting of sMIC with B10G5 enhances the efficacy of ACT for sMIC-producing tumors, we treated male B6/MICB mice bearing established syngeneic sMIC-producing B16F10-sMICB tumors as follows (Figure 1A): (1) isotype control mouse IgG (cIgG); (2) B10G5; (3) one-time Pmel ACT plus cIgG therapy twice weekly or (4) one-time Pmel ACT plus B10G5 therapy twice weekly.

**Figure 2:**
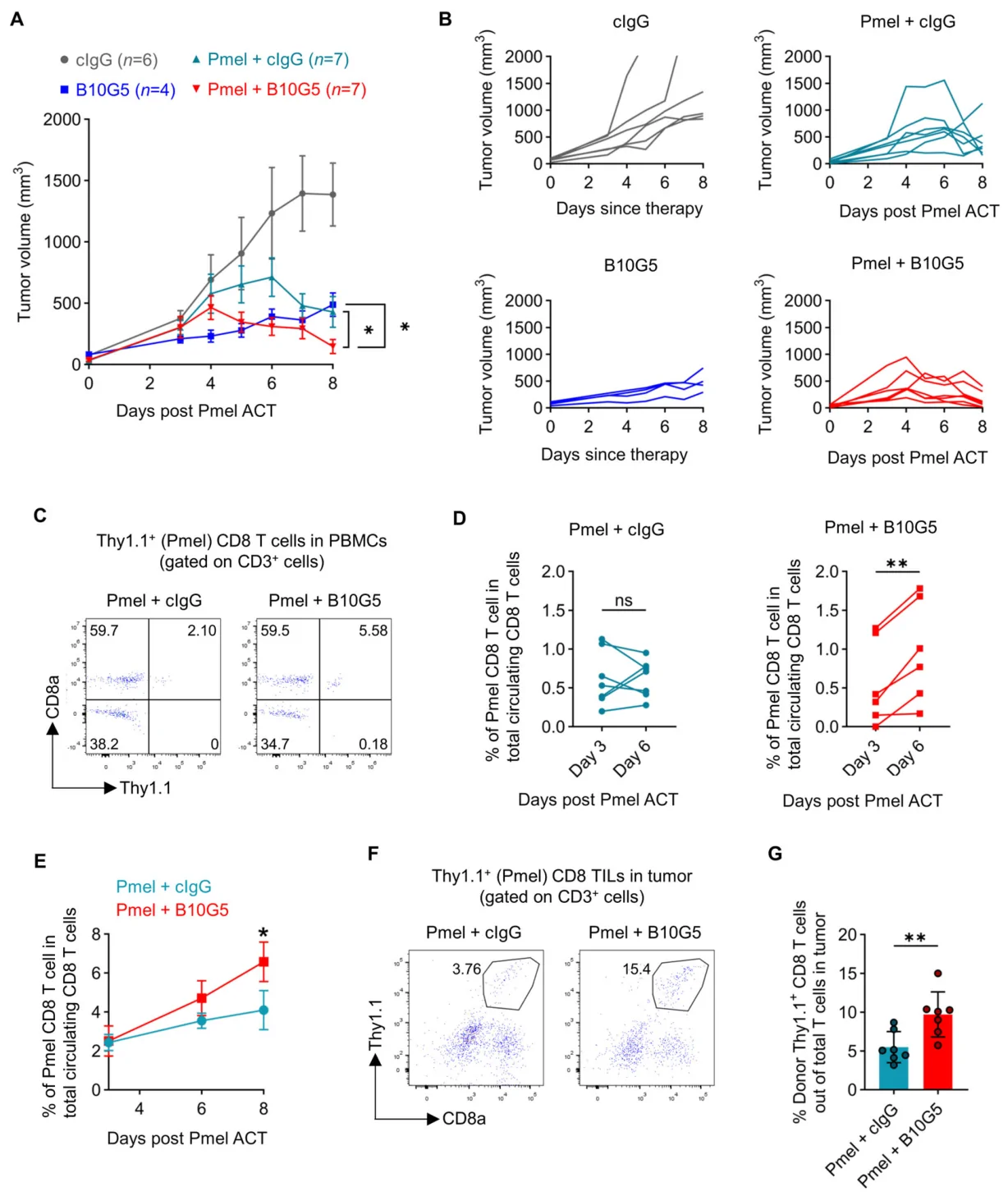
Antibody-targeting sMIC enhances the accumulation of donor CD8 T-cells in tumor and peripheral blood. (**A**) Tumor growth curves of treatment groups as indicated. Tumor volume at each time point was expressed as mean ± SEM in the respective treatment group. *, *p* < 0.05. p values indicated for either (1) B10G5 vs Pmel + B10G5, or (2) Pmel + cIgG vs Pmel + B10G5 comparisons. Statistical analysis was performed using Mann–Whitney tests: B10G5 vs Pmel + B10G5 (*p* = 0.0242; Mann–Whitney U-value = 2); Pmel + cIgG vs Pmel + B10G5 (*p* = 0.0379; Mann–Whitney U-value = 8). (**B**) Individual tumor growth curves in each therapy group. (**C**) Representative flow cytometry plots showing the frequency of donor Thy1.1^+^CD8^+^T cells in the circulation in treatment groups. (**D**) Dynamics of donor Thy1.1^+^CD8^+^ T cells in the circulation from day 3 to day 6 post-transfer. Note: Blood for one animal at day 6 of the Pmel +B10G5 group was lost during processing, and thus only data from 6 mice are shown. Pmel +cIgG, n = 7; two-tailed paired *t*-test: (*p* = 0.9899) (t = 0.01322, df = 6). Pmel + B10G5, n=6; two-tailed paired *t*-test: (*p* = 0.0062) (t = 4.527, df = 5). (**E**) Quantitative summary showing the dynamics of donor Thy1.1^+^CD8^+^ T-cells in the peripheral blood. (n = 7 for Pmel +cIgG; n = 6 for Pmel + B10G5). Day 8 comparison was performed using a two-tailed Mann–Whitney test: (*p* = 0.0379) (U = 8). (**F**,**G**) Representative flow cytometry plots (**F**) and quantitative summary (**G**) showing the frequency of donor Thy1.1^+^CD8^+^T cells at day 20 in tumors. Summary data are presented as mean ± SD. Individual data points represent a single animal. Statistical analysis was performed using a two-tailed unpaired Student’s *t*-test: (*p* = 0.0018) (t = 3.170, df = 12). *, *p* < 0.05; **, *p* < 0.01.

All therapies, whether B10G5 or Pmel ACT monotherapy or Pmel ACT in combination with B10G5, significantly inhibited tumor growth and extended overall survival of mice compared with the mouse IgG1 isotype control (cIgG) therapy (Figure 1B,C). Animals that received the combination therapy of B10G5 and Pmel ACT showed marked improvement in tumor control and overall survival in comparison to all other therapy groups (Figure 1B,C). Although therapy with Pmel ACT plus cIgG initially inhibited tumor growth in 3/7 animals, the therapeutic effect was not sustained, as aggressive tumor growth recurred by day 25 to 27 post-Pmel ACT (Figure 1C). Strikingly, three of five mice receiving Pmel ACT and B10G5 combination therapy had no detectable tumors by day 21 post-Pmel ACT (Figure 2C). These data demonstrate that therapeutic targeting of sMIC with B10G5 can markedly enhance the anti-tumor efficacy of Pmel ACT.

As described previously [47,50,52,53], B10G5 is a mouse IgG1 monoclonal antibody that mediates its immunomodulatory activity primarily by capturing sMIC, thereby neutralizing the immunosuppressive effects of sMIC on antitumor immunity. To further establish that the ability of B10G5 to enhance Pmel ACT is dependent on its targeting of sMIC rather than on a nonspecific antibody-mediated effect, we evaluated B10G5 in mice bearing established sMIC-negative B16F10 tumors. Mice received treatment with Pmel ACT alone or in combination with B10G5. In this sMIC-negative tumor setting, B10G5 provided no significant additional therapeutic benefit over Pmel ACT alone (Supplementary Figure S2). These findings demonstrate that the enhancement of Pmel-1 ACT by B10G5 requires the presence of sMIC and further support an sMIC-dependent mechanism of action for B10G5.

### 3.2. Targeting sMIC with B10G5 Enhances the Persistence and Tumor Accumulation of Adoptively Transferred Pmel CD8^+^ T-Cells

To investigate the underlying effector mechanisms associated with the improved treatment response of Pmel ACT by concurrent B10G5-targeting sMIC, we performed a “mechanistic-endpoint” experiment with a similar experimental schema as illustrated in Figure 1A, but the study was ended at day 8 post-Pmel ACT. Consistent with the survival study findings shown in Figure 1, Pmel ACT in combination with B10G5-targeting sMIC presented significantly greater tumor control than the monotherapy of B10G5 or Pmel ACT (Figure 2A,B). The transient aggressive tumor growth in mice that received Pmel and cIgG or Pmel and B10G5 prior to tumor regression was likely due to pseudo-progression induced by amplified immune responses at the tumor site [54]. To determine whether B10G5 therapy enhances the persistence of adoptively transferred Pmel CD8^+^T cells, we longitudinally monitored donor Pmel CD8^+^T cells in the peripheral blood and assessed their accumulation within tumors with an antibody specific to the congenic marker Thy1.1. In mice receiving Pmel-1 ACT and cIgG, the frequency of donor Thy1.1^+^CD8^+^T cells in peripheral blood remained largely unchanged between days 3 and 6; in contrast, mice receiving Pmel ACT in combination with B10G5 exhibited a significant increase in circulating Pmel CD8^+^T cells over the same period (Figure 2C,D). Consistent with this finding, the frequency of donor Thy1.1^+^CD8^+^T cells among peripheral CD8^+^T cells was significantly higher in the B10G5 combination treatment group on day 8 after Pmel ACT (Figure 2E).

We next examined the presence of donor Pmel CD8^+^T cells within the tumor. B10G5 treatment significantly increased the frequency of donor Thy1.1^+^CD8^+^T cells among tumor-infiltrating CD8^+^T cells compared with Pmel ACT alone (Figure 2F,G). Together, these findings demonstrate that targeting sMIC with B10G5 promotes the peripheral persistence of adoptively transferred Pmel CD8^+^T cells and enhances their accumulation within the tumor, providing a potential cellular basis for the improved antitumor efficacy of the combination therapy.

### 3.3. Targeting sMIC with B10G5 Enhances the Antigen-Specific Effector Function of Donor Pmel Cells

We next investigated whether targeting sMIC with B10G5 enhances the antigen-specific functional capacity of adoptively transferred Pmel T-cells. Splenocytes were isolated from B16F10-sMICB tumor-bearing B6/MICB mice treated with Pmel ACT in combination with either control cIgG or B10G5. The isolated splenocytes were seeded into IFN-γ ELISpot plates and stimulated overnight ex vivo with the Pmel-1 cognate antigen gp100_25_-_33_ peptide.

**Figure 3:**
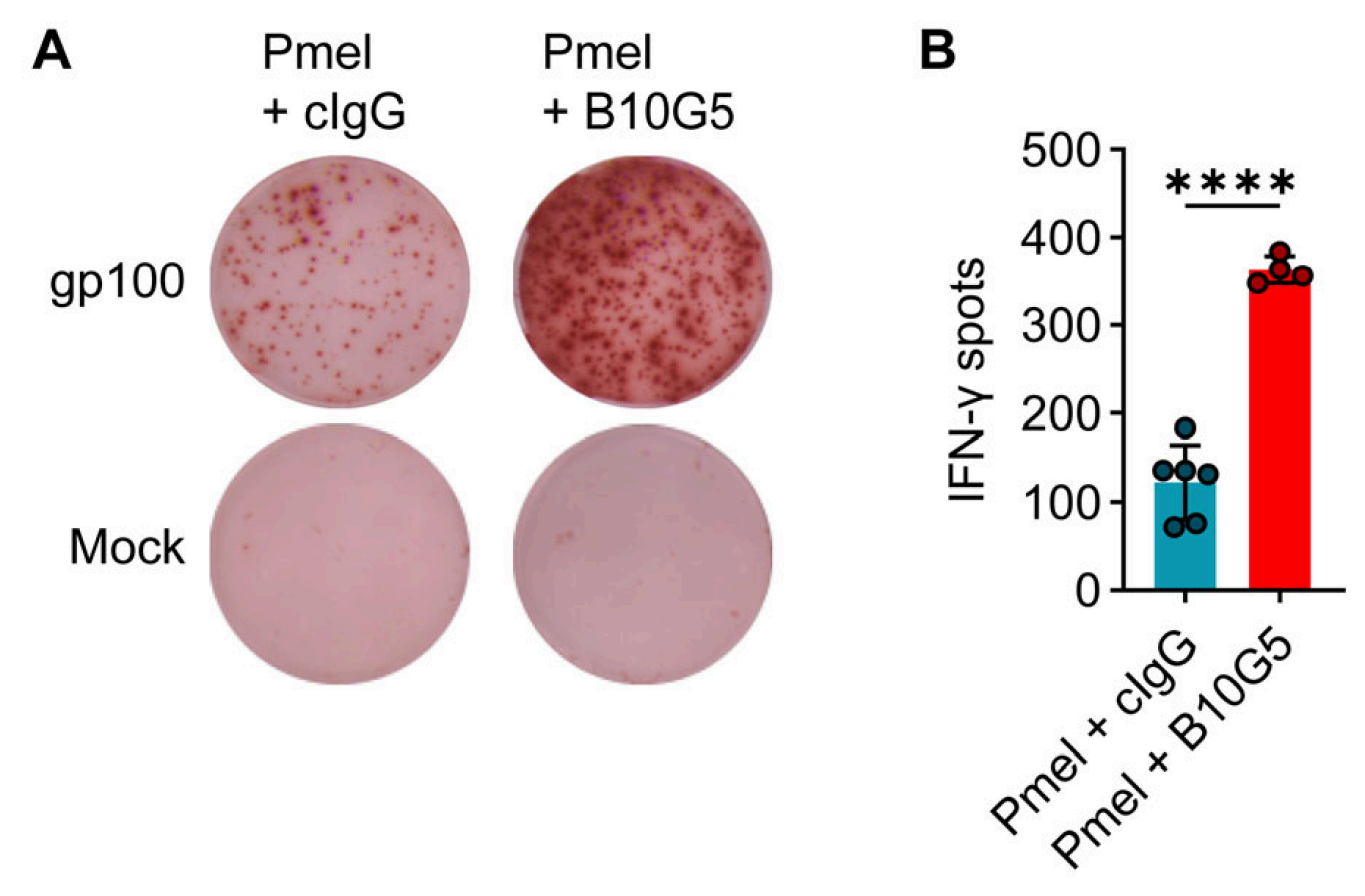
B10G5 targeting sMIC enhances antigen-specific effector responses of donor Pmel T-cells. (**A**) Representative IFN-γ ELISpot images of Pmel splenocytes isolated from B6/MICB mice bearing B16F10-sMICB tumors and treated with either Pmel ACT plus control cIgG or Pmel ACT plus B10G5. Cells were stimulated in ELISpot plates with 1 μM gp100_25−33_ overnight. Unstimulated (“mock”) Pmel splenocytes were used as a negative control. (**B**) Quantification of IFN-γ spot-forming cells followed. n = 6 for Pmel + cIgG; n = 4 for Pmel + B10G5. **** = *p* < 0.0001. Statistical analysis was performed using a two-tailed unpaired Student’s *t*-test: (*p* < 0.0001) (t = 10.71, df = 8). Data are presented as mean ± SD. Individual data points represent a single animal.

Splenocytes from mice receiving B10G5 in combination with Pmel ACT exhibited a markedly greater IFN-γ response to gp100_25-33_ stimulation than those from mice receiving Pmel ACT plus cIgG, as demonstrated by a significant increase in IFN-γ spot-forming cells (Figure 3A,B). These findings indicate that, in addition to enhancing the persistence and tumor accumulation of adoptively transferred Pmel T-cells, B10G5 treatment is associated with enhanced antigen-specific effector function, supporting improved functional competence of donor Pmel T-cells during sMIC-targeted therapy.

### 3.4. NK Cells Are Required for Optimal B10G5-Enhanced Antitumor Efficacy and Intratumoral Accumulation of Adoptively Transferred Pmel-1 CD8^+^ T-Cells

**Figure 4:**
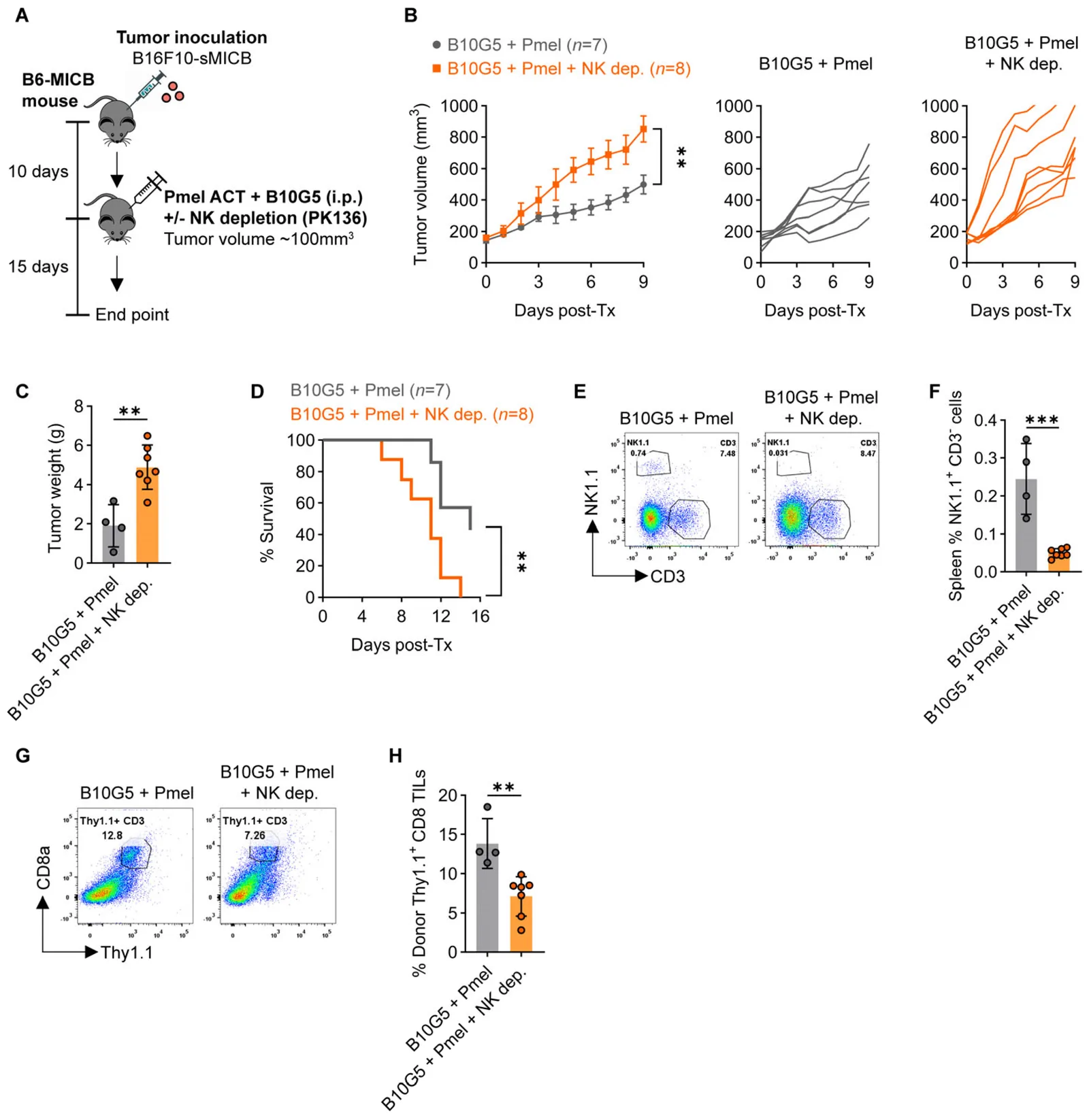
NK cell depletion diminishes the therapeutic efficacy of combined Pmel ACT and sMIC-targeting with B10G5. (**A**) Experimental design. B6/MICB male mice (n = 6–8 per group) were inoculated subcutaneously in the right flank with 4 × 10^5^ B16F10-sMICB cells and treated with: (1) B10G5 (3 mg/kg, i.p.) in combination with Pmel ACT (5 × 10^6^/mouse) (indicated in grey) or (2) B10G5 + Pmel in the setting of NK cell depletion with anti-NK1.1 antibody (clone PK136; 100 µg/mouse, i.p.). (indicated in orange) (**B**) Mean (left, mean ± SEM) and individual (middle and right) tumor growth curves of B16F10-sMICB tumors in the indicated treatment groups. Statistical analysis of day 9 tumor volume was performed using two-tailed unpaired Student’s *t*-test (*p* = 0.0054) (t = 3.332, df = 13). (**C**) Final tumor weights (mean ± SD) on day 15 in mice treated with B10G5 + Pmel (n = 4) or B10G5 + Pmel + NK cell depletion (n = 7). Statistical analysis was performed using two-tailed unpaired Student’s *t*-test: (*p* = 0.002, t = 4.304, df = 9). (**D**) Kaplan–Meier survival curves of B16F10-sMICB tumor-bearing mice receiving the indicated therapies. Statistical analysis was performed using Mantel–Cox Log-rank test. Chi square = 6.726; *p* = 0.0095. (**E**,**F**) Representative flow cytometry plots (**E**) and quantitative summary (**F**) confirming the effectiveness of NK depletion in splenocytes isolated from treated mice at study endpoint. ***, *p* < 0.001. n = 4, B10G5 + Pmel; n = 7, B10G5 + Pmel + NK depletion. Statistical analysis was performed using two-tailed unpaired Student’s *t*-test. (*p* = 0.0003, t = 5.697, df = 9). (**G**,**H**) Representative flow cytometry plots (**G**) and quantitative summary (**H**) showing the frequency of donor Thy1.1^+^ CD8^+^ T-cells in tumor on day 15 post-Pmel transfer. Individual data points represent individual animals. **, *p* < 0.01. Statistical analysis was performed using two-tailed unpaired Student’s *t*-test: (*p* = 0.0035, t = 3.924, df = 9). Individual data points represent each individual animal. Tx, Pmel ACT.

Lymphodepletion prior to ACT is standard practice, partially based on studies in Rag-deficient hosts showing that NK cells may limit ACT efficacy by competing for cytokines [55]. We thus investigated the contribution of NK cells to the enhanced therapeutic efficacy of Pmel-1 ACT in combination with B10G5. Cohorts of B16F10-sMICB tumor-bearing mice were randomized into two treatment groups: (1) Pmel-1 ACT in combination with B10G5 (3 mg/kg) or (2) Pmel-1 ACT in combination with B10G5 (3 mg/kg) in the setting with the addition of anti-NK1.1 antibody PK136 (3 mg/kg, twice weekly to study endpoint) to deplete NK cells (Figure 4A). The depletion efficiency was confirmed at the study endpoint by flow cytometry analysis of the NK cell population in the spleens with anti-CD3 and anti-NKp46 antibodies (Supplementary Figure S3).

NK cell depletion markedly compromised the therapeutic efficacy of the B10G5/Pmel combination, resulting in accelerated tumor growth over the 15-day observation period (Figure 4B), and significantly increased final tumor weight (Figure 4C). NK cell depletion also shortened survival to the predefined tumor-volume endpoint (Figure 4D). Flow cytometric analysis confirmed effective NK cell depletion, as the frequency of splenic NK1.1^+^CD3^−^ cells was markedly reduced in PK136-treated mice compared with mice receiving B10G5/Pmel therapy without PK136 (Figure 4E,F).

We next examined whether NK cell depletion affected the donor Pmel CD8^+^T cell response within the tumor. The frequency of donor Thy1.1^+^CD8^+^T cells among tumor-infiltrating CD8^+^ T-cells was significantly reduced following NK cell depletion (Figure 4G,H). Thus, the loss of NK cells was associated with both diminished intratumoral accumulation of donor Pmel CD8^+^T cells and impaired tumor control. Collectively, these findings demonstrate that NK cells are critical for the optimal therapeutic efficacy of Pmel ACT and sMIC-targeting combination therapy and suggest that NK cells support the maintenance and/or accumulation of adoptively transferred tumor-specific CD8^+^ T-cells within the tumor microenvironment.

### 3.5. NK Cells Are Required for Optimal Activation and Effector Function of Adoptively Transferred Antigen-Specific CD8^+^ T-Cells During sMIC-Targeted Therapy

**Figure 5:**
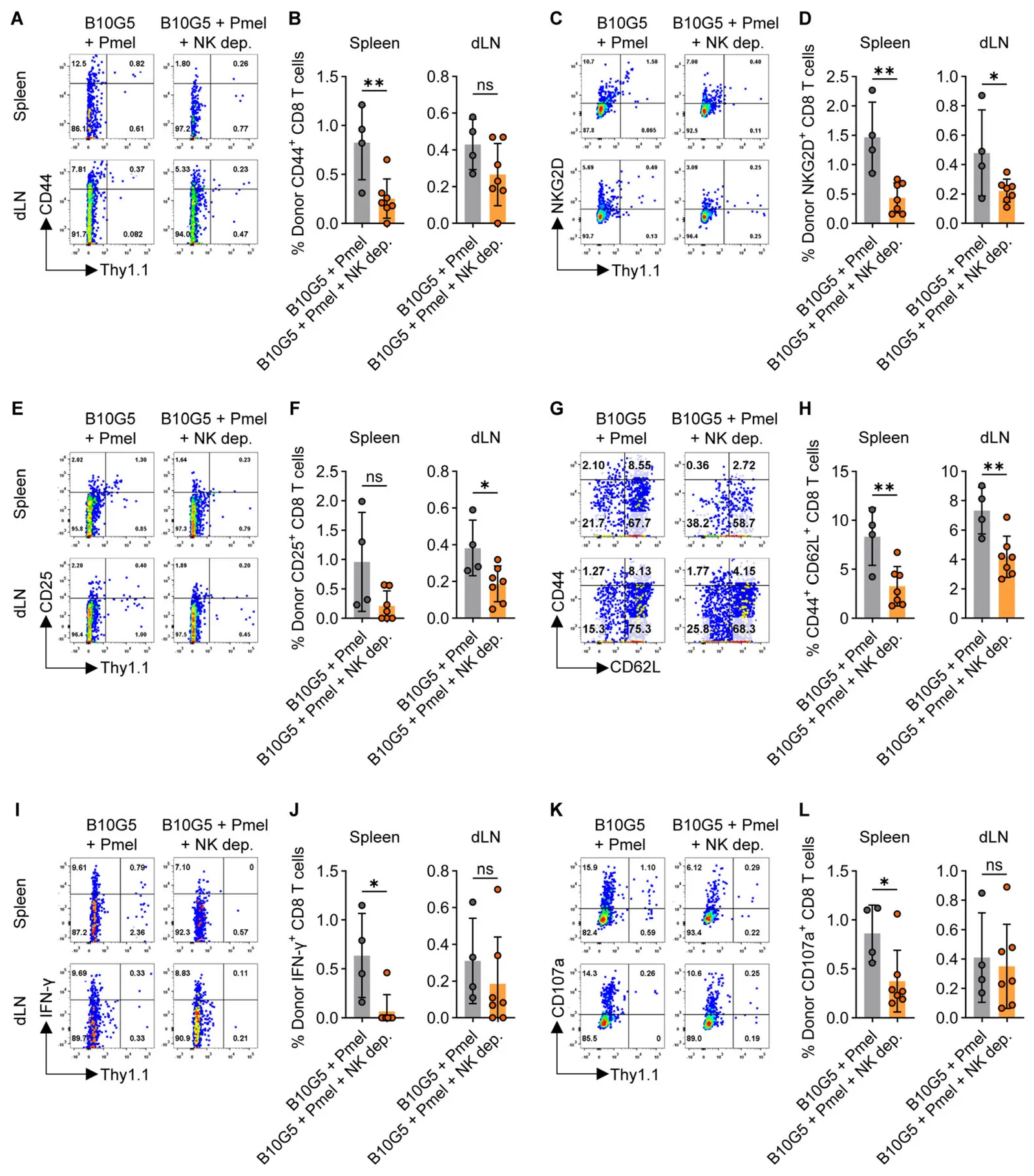
NK cell depletion impairs peripheral activation and effector function of donor Pmel CD8^+^T cells and sMIC-targeting combination therapy. (**A**,**B**) Representative flow cytometry plots (**A**) and quantitative summary (**B**) showing the frequency of activated donor Thy1.1^+^CD44^+^CD8^+^ T cells from spleen and dLNs of treatment groups. For (**B**), statistical analysis was performed using two-tailed unpaired Student’s *t*-test. For spleens, *p* = 0.0087, t = 3.339, df = 9; for dLN, *p* = 0.134, t = 1.647, df = 9. (**C**,**D**) Representative flow cytometry plots (**C**) and quantitative summary (**D**) showing NKG2D expression in Thy1.1^+^ donor CD8^+^T cells from spleen and dLNs of treatment groups. For (**D**), statistical analysis was performed using two-tailed unpaired Student’s *t*-test. For spleens, *p* = 0.0028, t = 4.079, df = 9; for dLN, *p* = 0.049, t = 2.275, df = 9. (**E**,**F**) Representative flow cytometry plots (**E**) and quantitative summary (**F**) showing the frequency of donor Thy1.1^+^ CD25^+^ CD8^+^T cells from spleen and dLN. Statistical analysis was performed using two-tailed unpaired Student’s *t*-test. For spleen, *p* = 0.0501, t = 2.261, df = 9. For dLN, *p* = 0.0268, t = 2.642, df = 9. (**G**,**H**) Representative flow cytometry plots (**G**) and quantitative summary (**H**) showing CD44^+^ CD62L^+^ memory CD8^+^T cells from spleen and dLNs in treatment groups as indicated. Statistical analysis was performed using two-tailed unpaired Student’s *t*-test. For spleen, *p* = 0.0076, t = 3.424, df = 9. For dLN, *p* = 0.0078, t = 3.410, df = 9. (**I**,**J**) Representative flow cytometry plots (**I**) and quantitative summary (**J**) showing the frequency of donor Thy1.1^+^ IFN-γ^+^ CD8^+^T cells in spleen and dLN of treatment groups. Statistical analysis was performed using two-tailed unpaired Student’s *t*-test. For spleen, *p* = 0.0108, t = 3.202, df = 9. For dLN, *p* = 0.444, t = 0.8006, df = 9. (**K**,**L**) Representative flow cytometry plots (**K**) and quantitative summary (**J**) showing the frequency of donor CD107a^+^ Thy1.1^+^ donor CD8^+^T cells from spleen and dLN of treatment groups. Cells were stimulated with gp100. Individual data points represent a single animal. Statistical analysis was performed using two-tailed unpaired Student’s *t*-test. For spleen, *p* = 0.0323, t = 2.529, df = 9. For dLN, *p* = 0.7525, t = 0.3252, df = 9. All summary data in bar graphs are presented as mean ± SD. For all bar graphs, B10G5 + Pmel, n = 4; B10G5 + Pmel + NK dep., n = 7. *, *p* < 0.05; **, *p* < 0.01; ns, not significant (*p* ≥ 0.05).

We investigated whether NK cell depletion alters the activation and effector function of adoptively transferred Pmel CD8^+^T cells in the B16F10-sMICB tumor model. NK cell depletion significantly reduced the frequencies of splenic donor Pmel CD8^+^T cells expressing the activation-associated markers CD44 (Figure 5A,B) and NKG2D (Figure 5C,D). In tumor-draining lymph nodes (dLNs), NK cell depletion similarly reduced the frequencies of donor Pmel CD8^+^T cells expressing NKG2D (Figure 5C,D) and CD25 (IL-2Rα) (Figure 5E,F). These findings indicate that NK cells contribute to maintaining the activated phenotype of adoptively transferred tumor-specific CD8^+^T cells during sMIC-targeting B10G5 combination therapy.

We next examined the memory phenotype of CD8^+^T cells. The frequency of CD44^+^CD62L^+^ memory-phenotype CD8^+^T cells, encompassing both host and donor populations, was significantly reduced in the spleens and dLNs of NK-depleted mice compared with mice receiving Pmel ACT and B10G5 combination therapy without NK depletion (Figure 5G,H), suggesting that NK cells also contribute to the maintenance of a memory-like CD8^+^T cell population.

To determine whether NK cell depletion also affected CD8^+^T cell effector function, we assessed cytokine production and degranulation. Following ex vivo stimulation with PMA/ionomycin, the frequency of IFN-γ-producing donor Pmel CD8^+^T cells was significantly reduced in the spleens of NK-depleted mice (Figure 5I,J). Furthermore, following ex vivo stimulation with the Pmel cognate antigen gp100, NK cell depletion significantly reduced the frequency of CD107a^+^ donor Pmel CD8^+^T cells (Figure 5K,L), indicating impaired antigen-specific degranulation.

Collectively, these findings demonstrate that NK cells contribute to sustaining the activation, memory phenotype, and effector competence of adoptively transferred Pmel CD8^+^T cells in combination with sMIC-targeting B10G5 therapy. Thus, the impaired tumor control observed following NK cell depletion is associated not only with reduced intratumoral accumulation of donor Pmel cells but also with diminished activation and functional capacity of the adoptively transferred tumor-specific CD8^+^T cells.

### 3.6. NK Cells Are Required for Optimal CD8^+^ T-Cell Function During B10G5/sMIC Stimulation

We previously demonstrated that B10G5 forms a complex with sMIC that engages NKG2D and promotes the activation and effector function of NK cells and CD8^+^T cells [52,53]. To determine whether NK cells directly contribute to enhanced CD8^+^T cell function in the context of B10G5/sMIC stimulation, we performed ex vivo proof-of-concept NK cell depletion experiments using splenocytes from B6 mice. Because naïve murine CD8^+^T cells do not constitutively express NKG2D, splenocytes were stimulated with anti-CD3 and anti-CD28 to activate CD8^+^T cells and induce NKG2D expression, thereby allowing assessment of the NKG2D^+^ activated CD8^+^T cell population.

**Figure 6:**
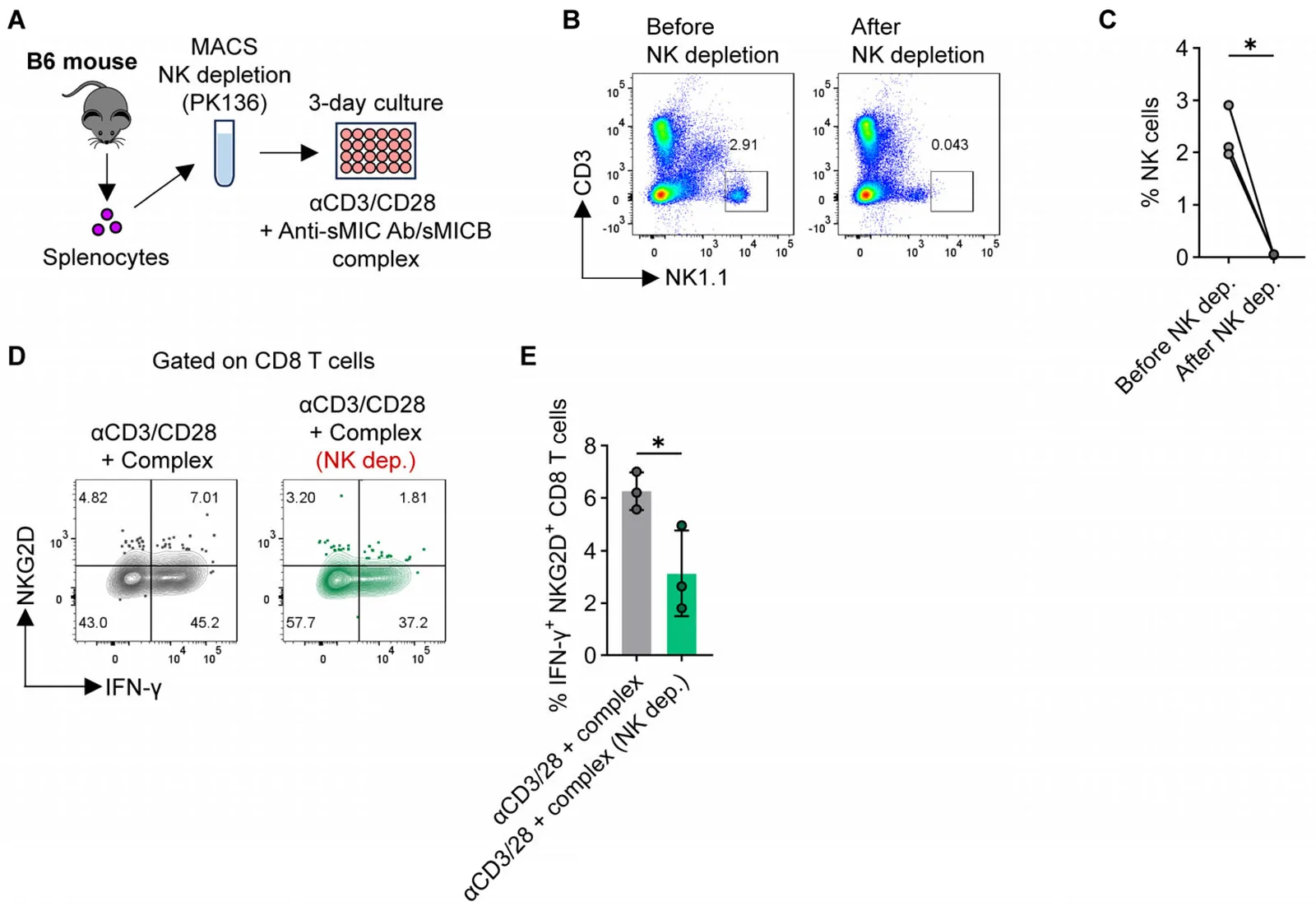
NK depletion from ex vivo splenocyte culture results in impaired CD8^+^T cell function in the context of B10G5/sMIC stimulation. (**A**) Experimental design. Splenocytes isolated from wild-type B6 mice were depleted of NK cells via magnetically activated cell sorting (MACS), using an anti-NK1.1-biotin (clone PK136) antibody incubated with Streptavidin nanobeads. Splenocyte samples with or without NK depletion were plated and stimulated with 1 μg/mL anti-CD3, 1 μg/mL anti-CD28, and 100 ng/mL B10G5/sMICB complex for 3 days. (**B**,**C**) Representative flow cytometry plots (**B**) and summary (**C**) of splenocytes from three mice showing efficiency of NK depletion. Statistical analysis was performed with paired *t*-test, *p* = 0.0018, t = 23.52, df = 2. (**D**,**E**) Representative flow cytometry plot (**D**) and summary data (**E**) showing that NK depletion impaired IFN-γ production by NKG2D^+^CD8^+^T cells. Statistical analysis was performed with paired *t*-test, *p* = 0.0383, t = 3.042, df = 4. *n* = 3 per group. Summary data are presented as Mean ± SD. Individual data points represent a single animal. *, *p* < 0.05.

NK-intact and NK-depleted splenocytes were stimulated with anti-CD3/anti-CD28 and cultured in the presence of B10G5/sMICB complex for 3 days (Figure 6A–C). CD8^+^T cell function was subsequently assessed by measuring IFN-γ production within the NKG2D^+^CD8^+^T cell population. NK cell depletion significantly reduced the frequency of IFN-γ-producing NKG2D^+^CD8^+^T cells (Figure 6D,E), demonstrating that the presence of NK cells is important for achieving an optimal CD8^+^T cell effector response under B10G5/sMICB stimulation. Together with the in vivo NK cell depletion studies, these findings support a functional interplay between NK cells and CD8^+^T cells during B10G5 therapy and suggest that NK cells contribute directly to sustaining the functional competence of activated CD8^+^T cells in the context of B10G5/sMIC engagement.

## 4. Discussion

In the present study, we demonstrate that therapeutic targeting of tumor-derived sMIC with B10G5 substantially enhances the antitumor efficacy of antigen-specific CD8^+^T cell adoptive cell therapy (ACT). Although adoptively transferred Pmel CD8^+^T cells alone exerted moderate control of tumor growth in the sMIC-producing B16F10 melanoma model, combination with B10G5 resulted in markedly improved tumor control, accompanied by increased persistence and intratumoral accumulation of donor Pmel cells and enhanced antigen-specific effector function. Importantly, depletion of NK cells substantially diminished the therapeutic benefit of the B10G5/Pmel combination and was associated with reduced donor CD8^+^T cell accumulation, activation, cytokine production, and degranulation. Together, these findings identify NK cells as important contributors to the enhanced efficacy of ACT during sMIC-targeted therapy and support a model in which NK and CD8^+^T cells function cooperatively to promote antitumor immunity.

To our knowledge, the efficacy of adoptively transferred Pmel cells has not previously been directly evaluated in the context of an sMIC-producing tumor. Our findings indicate that the therapeutic activity of Pmel ACT alone is limited in the presence of tumor-derived sMIC and that targeting this immunosuppressive pathway can substantially improve ACT efficacy. Previous studies using murine melanoma models have shown that the efficacy of Pmel ACT can be enhanced by lymphodepletion, in part through elimination of immunosuppressive cell populations and alteration of the cytokine environment available to transferred T-cells [55,56]. Regulatory T-cells and myeloid-derived suppressor cells (MDSCs), among other endogenous populations, are well-established contributors to immunosuppression within the tumor microenvironment [57]. Our findings identify therapeutic targeting of sMIC as a complementary strategy for improving ACT by modifying a distinct immunosuppressive pathway rather than relying solely on broad depletion of endogenous immune populations.

A particularly important finding of this study is the contribution of NK cells to the therapeutic efficacy of the B10G5/Pmel combination. NK cell depletion markedly impaired tumor control and reduced the accumulation of donor Pmel CD8^+^T cells within tumors. Moreover, NK depletion was associated with reduced expression of activation-associated markers on donor Pmel cells, diminished IFN-γ production and antigen-specific degranulation, and reduced frequencies of CD44^+^CD62L^+^ memory-phenotype CD8^+^ T-cells in secondary lymphoid organs. These observations indicate that, in the setting of sMIC-targeted therapy, NK cells contribute not only through their intrinsic antitumor activity but also by supporting the magnitude and functional competence of the CD8^+^T cell response.

The beneficial contribution of NK cells observed here differs from reports in which NK cells can compete with adoptively transferred CD8^+^T cells for homeostatic cytokines and thereby limit T-cell expansion [55]. An important distinction is that some of these studies were performed under profoundly lymphopenic conditions, including Rag-deficient hosts, in which the relative abundance and cytokine consumption of NK cells differ substantially from those in immunocompetent hosts. Thus, the functional relationship between NK cells and transferred CD8^+^T cells is likely to depend on the immunological context. Rather than functioning exclusively as competitors for cytokines, NK cells may provide supportive signals that promote CD8^+^T cell activation, survival, differentiation, or effector function under appropriate conditions. Our findings therefore suggest that preservation of NK cell function may be particularly important when ACT is combined with therapies that engage the NKG2D axis.

Our findings also have implications for the use of lymphodepletion in ACT. Lymphodepletion is an established strategy for enhancing transferred T cell expansion and function through multiple mechanisms, including reduction of immunosuppressive populations and increased availability of homeostatic cytokines [56,57]. Our results do not argue against lymphodepletion per se; rather, they suggest that the extent and composition of lymphodepletion may be important when the therapeutic strategy depends on cooperation between transferred T cells and endogenous NK cells. Approaches that preserve or restore functionally competent NK cells while reducing suppressive immune populations could potentially maximize the benefit of sMIC-targeted ACT. This concept warrants further investigation in clinically relevant models.

Although we focused primarily on adoptively transferred Pmel cells, modulation of the sMIC–NKG2D axis may also affect endogenous tumor-reactive CD8^+^T cell responses. We did not directly quantify endogenous tumor-antigen-specific CD8^+^T cells in the present study and therefore cannot determine whether B10G5 increases their abundance or +function. Nevertheless, the enhanced activation and effector environment generated by sMIC targeting could potentially support endogenous as well as transferred tumor-reactive T-cells. The magnitude and quality of such responses may depend on precursor frequency, antigen affinity, and intra-clonal competition, all of which can influence effector expansion and memory differentiation [58,59,60]. Future studies distinguishing endogenous from transferred tumor-reactive populations will be necessary to define their respective contributions to the therapeutic response.

Several limitations should also be considered. Although NK cell depletion established a substantial contribution of NK cells to the therapeutic response, other NKG2D-expressing lymphocyte populations, including NKT cells and γδ T-cells, may also participate [61]. In addition, the mechanisms through which B10G5 supports NK and CD8^+^T cell function are likely multifactorial. Beyond the activity of B10G5/sMIC complexes, targeting soluble MIC may limit chronic sMIC-mediated NKG2D downregulation and thereby preserve NKG2D-dependent responsiveness of NK and CD8^+^T cells. B10G5 may also reshape myeloid cell function in the tumor microenvironment. B10G5 therapy may also enhance DC and macrophage antigen presentation capacity in the TME through cell–cell crosstalk and immune-stimulatory cytokine and chemokine secretion by NK cells. The relative contribution of each of these cell types and components requires systemic studies.

Overall, our findings demonstrate that targeting tumor-derived sMIC can substantially enhance antigen-specific CD8^+^T cell ACT and reveal an important role for NK cells in sustaining the persistence, activation, and effector function of transferred T-cells. These results support a model in which sMIC-targeted therapy promotes coordinated NK cell and CD8^+^ T cell antitumor immunity rather than acting solely through independent enhancement of either lymphocyte population. Given the prevalence of sMIC in human malignancies and its absence from conventional murine models, these findings provide a rationale for further development of sMIC-targeting strategies in combination with adoptive T-cell therapies for sMIC^+^ solid tumors.

## 5. Conclusions

In summary, our study identifies therapeutic targeting of tumor-derived sMIC as a strategy to substantially enhance the efficacy of antigen-specific CD8^+^T cell adoptive cell therapy against sMIC^+^ solid tumors. Importantly, our findings reveal a critical role for NK cells in supporting the therapeutic efficacy of the combination treatment. NK cells promote the persistence, intratumoral accumulation, activation, and effector function of adoptively transferred CD8^+^ T-cells, highlighting a previously underappreciated functional cooperation between NK cells and tumor-specific CD8^+^T cells during sMIC-targeted therapy. Collectively, these findings provide preclinical proof-of-concept for combining sMIC-targeting antibodies with adoptive T-cell therapy and establish NK cell andCD8^+^T cell cooperation as an important component of effective antitumor immunity within the immunosuppressive tumor microenvironment.

## Data Availability

Data sharing is available upon request.

## Supplementary Materials

The following supporting information can be downloaded at: https://www.mdpi.com/article/10.3390/biomedicines14102390/s1, Figure S1. Flow cytometry confirmation of sMICB expression in B16F10-GFP cells. Sorted B16F10-GFP or B16F10-sMICB-GFP cells were incubated with BD Fix/Perm buffer, incubated mouse Fc blocker, followed by incubation with 1 μg/ml of anti-sMIC/MIC monoclonal antibody (mouse IgG1) and PE-conjugated goat anti-mouse antibody. Figure S2. B10G5 has no significant impact on Pmel ACT in treating sMIC-negative B16F10 tumors. Cohorts of B6/MICB male mice were s.c. inoculated with B16F10 tumor cells (4 × 10^5^ cells/mouse). When tumors were established, mice were randomized into two treatment groups: (1) Pmel ACT (n = 7); (2) Pmel ACT + B10G5 (n = 6). Similar to treatment given to mice bearing B16F10-sMICB tumors, Pmel cells were activated for 3 days in the presence of gp100 and IL-12 before being used for ACT and were given one time via i.v. injection (5 × 10^6^/mouse). B10G5 was given one day prior to ACT (3 mg/kg) and thereafter twice weekly with the same dose during the experiment. (A) Tumor growth dynamics of each therapy group as indicated. Tumor volume was expressed as mean ± SD. ns, no significant difference statistically. Statistical analysis was performed using 2-way repeated measures ANOVA followed by Šídák’s multiple comparisons test: Column factor *p*-value (*p* = 0.8874) (F(1, 11) = 0.021). (B) Individual tumor growth dynamic in each therapy group. Figure S3. Flow cytometry confirmation of NK cell depletion in mice received PK136. Splenocytes from mice in respective treatment group (Pmel + B10G5 + PK136 and Pmel + B10G5) was stained with anti-mouse CD3 and anti-mouse NKp46 antibody. NK cells are gated as CD3^−^NKp46^+^ population. The data confirms the depletion of NK cells by PK136. Analysis was done at the study endpoint.
